# The effect of omega-3 fatty acids on alcohol-induced damage

**DOI:** 10.3389/fnut.2023.1068343

**Published:** 2023-04-05

**Authors:** Maitane Serrano, Irantzu Rico-Barrio, Pedro Grandes

**Affiliations:** ^1^Laboratory of Ultrastructural and Functional Neuroanatomy of the Synapse, Department of Neurosciences, Faculty of Medicine and Nursing, University of the Basque Country UPV/EHU, Leioa, Spain; ^2^Achucarro Basque Center for Neuroscience, Science Park of the UPV/EHU, Leioa, Spain

**Keywords:** polyunsaturated fatty acids, n-3, ethanol, brain damage, liver disease

## Abstract

Alcohol is the most widely consumed psychoactive substance in the world that has a severe impact on many organs and bodily systems, particularly the liver and nervous system. Alcohol use during pregnancy roots long-lasting changes in the newborns and during adolescence has long-term detrimental effects especially on the brain. The brain contains docosahexaenoic acid (DHA), a major omega-3 (n-3) fatty acid (FA) that makes up cell membranes and influences membrane-associated protein function, cell signaling, gene expression and lipid production. N-3 is beneficial in several brain conditions like neurodegenerative diseases, ameliorating cognitive impairment, oxidative stress, neuronal death and inflammation. Because alcohol decreases the levels of n-3, it is timely to know whether n-3 supplementation positively modifies alcohol-induced injuries. The aim of this review is to summarize the state-of-the-art of the n-3 effects on certain conditions caused by alcohol intake, focusing primarily on brain damage and alcoholic liver disease.

## 1. Introduction

Alcohol is a worldwide-consumed drug and its use depends on gender, age and health status. In 2016, 43.0% of the global adult population were current drinkers and caused 5.3% of all deaths (1). The average intake in the world is 13.9 grams of pure alcohol per day. Its use is more common and the consumption is the highest in the European Region (59.9%), followed by the Region of the Americas (54.1%) and the Western Pacific Region (53.8%).

Chronic heavy consumption of alcohol causes injuries in the organism, notably in the digestive, immune and central nervous system (CNS). The liver alcohol dehydrogenase metabolizes the acetaldehyde of alcohol making this organ particularly susceptible to lesion (2). Alcohol intake during pregnancy can result in damage in newborns with detrimental effects on memory (3) due to hippocampal and prefrontal cortical alteration (3, 4). In addition, alcohol use commonly starts during adolescence disrupting brain maturation and key processes of development. Binge drinking during adolescence has a long-lasting impact on hippocampal neurogenesis increasing cell death (5, 6) as well as on parahippocampal and neocortical structures altering brain plasticity, cognition and behavior (7–10). Likewise, we observed deficits in recognition, spatial and associative memory in early adulthood after chronic ethanol intake during adolescence (11, 12). Cognitive impairment in adult brain after adolescent alcohol intake correlates with changes in white matter, disrupted gray matter (13), reduced hippocampal volume and low levels of brain-derived neurotrophic factor in the adult hippocampus (14, 15). Furthermore, loss of prefrontal gray matter associates with motor, emotional and memory deficits (16) and the blood flow is altered in prefrontal and parietal regions of the female brain (17). Glial cells are also responsive to the adolescent binge drinking insult through the TLR4/NFκB signaling cascade with the consequent inflammatory response and the long-term impaired behavior and cognition (18, 19). Moreover, because dysfunctional swelled astrocytes with much less cannabinoid CB1 receptors is a key feature in adult hippocampus upon cessation of adolescent binge drinking (20), astroglial anti-inflammatory response mediated by cannabinoid receptors in astrocytes (21) is likely altered in alcohol conditions.

The brain is mainly composed of lipids, particularly docosahexaenoic acid (DHA) (22:6n-3), a n-3 long-chain polyunsaturated FA (PUFA), and eicosapentaenoic acid (EPA, 20:5n-3) and, at lower levels, by the omega-6 (n-6) arachidonic acid (AA) (22). The content of DHA varies among regions and between neurons and glial cell types (22), is essential for membrane structure and function, membrane-associated protein function, cell signaling, gene expression and lipid production (23, 24), having potent anti-inflammatory effects (22). The detrimental impact of alcohol on DHA (25–28) impairs synaptic plasticity (29–31), particularly N-methyl-D-aspartate (NMDA)-dependent long-term potentiation (LTP) in the hippocampus (31) and medial prefrontal cortex (30), two brain regions enriched with DHA (22). Also, adequate EPA levels are needed for a proper acute behavioral response to alcohol (32).

Compelling evidences have shown that n-3 protects against some brain conditions, such as multiple sclerosis, depression or schizophrenia (33). In Alzheimer’s and Parkinson’s disease, n-3 FAs ameliorate cognitive impairment related to synaptic plasticity disturbance (34), diminish oxidative stress (34, 35) and reduce neuronal death (35). They also decrease inflammatory cytokine levels in various pathologies (35, 36).

## 2. Alcohol damage to the nervous system and omega-3

Alcohol use during pregnancy can result in fetal alcohol spectrum disorder (FASD), characterized by long-lasting physical, mental, behavioral and learning deficits due to damage of the CNS (37). In the 1990s, first pieces of evidence provided a link between n-3 and FASD, as proper n-3 intake, particularly DHA and EPA, ameliorated newborn brain and body weight reduction caused by alcohol (38, 39). All the abnormalities of the developing brain elicited by prenatal ethanol exposure (PNEE) imply behavioral changes. N-3 intake improves locomotion and, although not at all ages, anxiety-like behavior in FASD (39, 40). Furthermore, DHA reverses the PNEE deficits in somatosensory performance, social behavior and vocalization (41).

**FIGURE 1:**
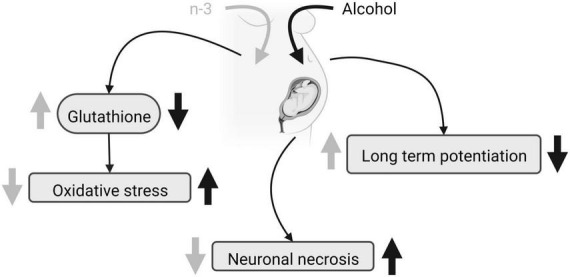
FASD and n-3 effects. Due to maternal ethanol intake, fetal glutathione decreases, oxidative stress augments, neuronal necrosis raises and LTP is impaired. Maternal n-3 enriched diet increases glutathione, decreases oxidative stress, reduces neuronal necrosis and rescues LTP, mitigating ethanol harmful effects. Created with www.Biorender.com.

Distinct protocols of ethanol administration result in different effects on brain phospholipid composition, ranging from a reduction in some saturated FA (12:0, 14:0, and 16:0) and a rise in 18:0 without diet effects (38), to unaffected levels of saturated FA (39). Furthermore, the ethanol-induced increase in n-6 and decrease in n-3 elevates the net n-6/n-3 ratio that can be reversed by a diet enriched in n-3 (38, 39, 42). N-3 increases the levels of 18:2n-6 and 20:3n-6 FA (38, 39), but more contradictory is the rise in 18:1n-9 achieved by ethanol intake (38) and a DHA-enriched diet (39). Although PNEE and diet do not alter the protein carbonyl levels detected by spectrophotometry, n-3 reduces lipid peroxidation in the dentate gyrus and prefrontal cortex after PNEE, thus preventing brain oxidative stress in FASD (43, 44). Furthermore, n-3 intake restores glutathione levels dampened by PNEE in adulthood (44), despite the fact that neither alcohol nor diet influence the activity of the superoxide dismutase and catalase. Glutathione regulates the thiol redox state of the cells positively affecting NMDA receptor function and plasticity disrupted by PNEE in the dentate gyrus of adult males, and recovered by a diet enriched with n-3 from birth to adulthood (45) (Figure 1). Also, n-3 lowers the increased caspase-3 and calpain activity detected by p-nitroanilin absorbance and calcium- and non-calcium-dependent fluorescence in ethanol conditions, reducing cell injury, neurodegeneration, brain hemorrhage, congestion, necrosis, leukocytosis and microglia activation (43).

**FIGURE 2:**
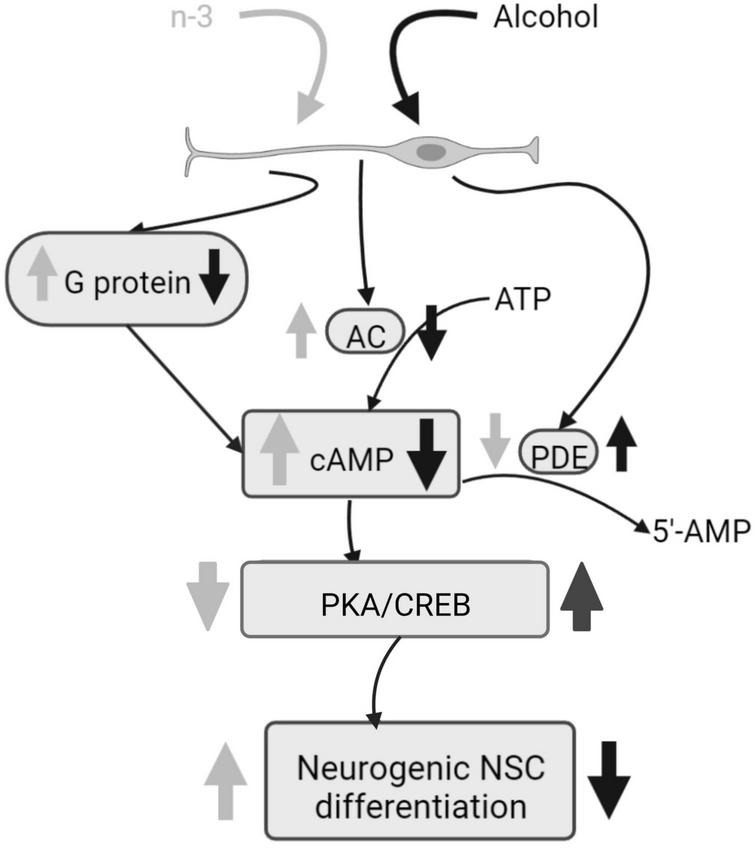
Alcohol damage of neurogenic NSC differentiation and n-3 effects. Alcohol decreases G protein and AC activation, consequently cAMP signaling is downregulated and PKA and CREB phosphorylation are increased, having a negative impact on NSC differentiation. PDE activity is upregulated by alcohol. However, n-3 intake improves cAMP signaling, decreasing PKA/CREB that promotes NSC differentiation. Also, n-3 downregulates PDE. Created with www.Biorender.com.

Chronic ethanol impairs both basal and forskolin induced cAMP-dependent neurogenic differentiation of neural stem cells (NSC), by decreasing adenylyl cyclase (AC) mRNA, phosphodiesterases (PDEs) activity, as well as by downregulating Cyclic adenosine monophosphate (cAMP) production by reducing G-protein activation analyzed by [γ-^35^S] GTP binding assays. Ethanol also reduces Tuj-1 expression, an early stage of neural differentiation marker, and protein kinase A (PKA) and cAMP-response element binding protein (CREB) phosphorylation. Noticeably, synaptamide, a DHA metabolite analog of the endocannabinoid anandamide, ameliorates cAMP signaling and boosts the NSC differentiation impaired by ethanol (46) (Figure 2).

Alcohol intake in adulthood promotes oxidative stress and neurodegeneration particularly in the hippocampus, entorhinal cortex and olfactory bulb (28, 47, 48). It also increases aquaporin-4 (AQP-4) involved in glial edema (28, 47), and poli ADP-ribose polymerase-1 (PARP-1) that favors necrosis through glial activation (28). In addition, a rise in phospholipase A2 (PLA2) which mobilizes AA from membrane phospholipids, a major reactive oxygen species (ROS) source in brain conditions, was revealed by immunoblotting and scintillation spectroscopy after binge ethanol (28, 48). Also, the ethanol-induced enhancement of 4-hydroxynonenal-adducted and 3-nitrotyrosinated (3NT) proteins, two oxidative footprints derived from AA and free radicals, were detected by immunoblot analysis (28). The ethanol-elicited DHA decrease shown by PI fluorescence labeling in cultures is detrimental, as this PUFA abolishes the changes in AQP-4, PLA2, PARP-1, and 3NT caused by alcohol with the consequent positive effects on neurodegeneration and oxidative stress (28, 47, 48). Likewise, DHA enrichment is able to counteract its own low brain levels due to alcohol (28).

## 3. Alcoholic liver disease and omega-3

*In vitro* investigations have shown opposite effects of the main n-3 FAs on hepatocyte damage caused by ethanol, with their more significant impact on lipid rafts (49, 50). Thus, EPA promotes membrane remodeling by phospholipase C (PLC) translocation into lipid rafts, enhancing the oxidative stress and cell death of ethanol that can be diminished by vitamin E and membrane stabilizers (49). In contrast, DHA prevents the harmful ethanol effects on hepatocytes by inhibiting PLC relocation into lipid rafts (50).

Oxidative stress causes membrane lipid peroxidation, impairs mitochondrial function and decreases antioxidant enzymatic activity, all together leading to liver damage (51). DHA mitigates PNEE-induced fetal liver enlargement and reduces alcohol rise of liver oxidative stress by normalizing the excess of glutathione reductase mRNA and the deficit in glutathione peroxidase mRNA, as detected by real-time quantitative polymerase chain reaction (52). Like in FASD, the increased liver-to-body weight ratio elicited by alcohol is reduced by n-3 in adulthood (53, 54). Ethanol increases ROS and reactive nitrogen species, thus rising lipid peroxidation measured by levels of hepatic malondialdehyde (55). The increase in CYP2E1 and, therefore, in ROS and acetaldehyde production (54, 55), as well as in inducible nitric oxide synthase that rises hydrogen peroxide and nitrite (55) point to the alcohol challenge of mitochondrial function. Also, conventional Oil Red O and hematoxylin and eosin staining revealed lipid droplets (54, 56, 57) and hepatocellular ballooning (54, 55) caused by alcohol disruption of the FA enzymatic metabolism (55). Compelling pieces of evidence have shown that n-3 reduces ROS (54, 55) and DHA supplementation promotes heme oxygenase-1 protein and mRNA against oxidative stress and cell death (54, 56).

**FIGURE 3:**
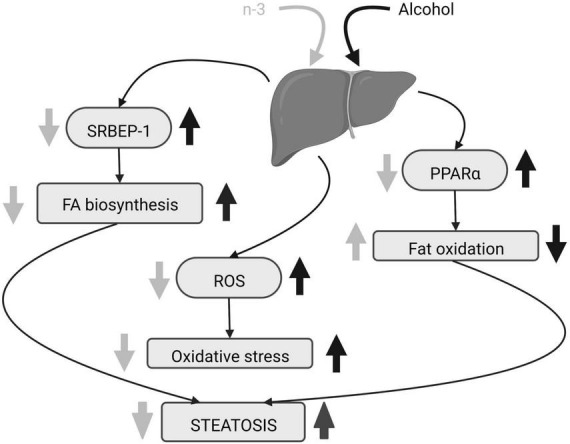
Alcohol-induced hepatic steatosis and n-3 effects. The increase in SRBEP-1 and ROS by alcohol elevates liver FA biosynthesis and oxidative stress, respectively, and alcoholic PPARα rise reduces liver fat oxidation. The consequence is steatosis. N-3 enriched diet lowers SRBEP-1 and ROS, decreasing liver FA biosynthesis and oxidative stress. It also promotes liver fat oxidation by downregulating activation of PPARα. Altogether, n-3 ameliorates steatosis. Created with www.Biorender.com.

N-3 reduces adipose lipolysis and FA biosynthesis (53). DHA binds to the G protein-coupled receptor 40 in hepatocytes downregulating the sterol regulatory element-binding protein 1 (SRBEP-1) that controls gene expression of lipogenic enzymes (54, 57, 58), thus decreasing triglycerides and cholesterol accumulation in liver caused by alcohol consumption (55, 57). Furthermore, n-3 diminishes gene expression and activation of lipolytic enzymes and promotes adipose tissue storage (53) as well as FA oxidation by activation of peroxisome proliferator-activated receptor alpha (PPARα) (54, 57). This results in the decrease in lipid droplets and hepatocellular ballooning ameliorating liver steatosis (55, 57, 59) (Figure 3). In fact, DHA supplementation combined with extra virgin olive oil prevents the fall of PPARα mRNA and its target genes in animals fed in a high fat diet to induce hepatic steatosis (60). The n-3 enrichment also decreases alanine aminotransferase, aspartate aminotransferase, alkaline phosphatase and total bilirubin, lowers n-6/n-3 ratio and suppresses ethanol liver inflammation by reducing pro-inflammatory cytokines (53, 54, 56, 59, 61). Finally, lipolysis increase, high free FA flow to the liver, pro-inflammatory cytokines rise, FA oxidation dysregulation and *de novo* lipogenesis, are common features in both ethanol-induced liver injury and non-alcoholic fatty liver disease (NAFLD) (62). All these changes have been linked to the existence of a chronic detrimental low-grade inflammatory state in the adipose tissue (62). Lipid mediators generated from EPA (E series resolvins) and DHA (D series resolvins, protectins, maresins) (62) have anti-inflammatory properties contrary to n-6 derivatives (59). In fact, current pieces of evidence indicate that the antioxidant, anti-inflammatory and anti-apoptotic effects of DHA on metabolic diseases, including NAFLD, are mediated by resolvins (63–65).

## 4. Other organs damaged by alcohol and the effects of omega-3

DHA alleviates testosterone fall due to early ethanol exposure and recovers the low steroidogenic acute regulatory protein mRNA caused by PNEE in adolescence, responsible for cholesterol transport into mitochondria needed for testosterone synthesis. Also, DHA increases sperm number and morphology in adulthood (66). Diets enriched in both DHA and EPA modify phospholipid FA composition in rat gastric mucosal cells (67). Likewise, n-3 enriched diet (but not EPA alone) reduces the stomach lesion due to alcohol-induced gastric hemorrhage. Combined exposure to ethanol and palmitoleic acid (POA) increases intracellular and mitochondrial ROS in AR42J pancreatic cells that results in a significant increase in the necroptosis mediator receptor-interacting protein and mixed lineage kinase domain-like pseudokinase. EtOH/POA also promotes calcium overload and nicotinamide adenine dinucleotide phosphate hydrogen (NADPH) oxidase activation. However, DHA suppresses EtOH/POA-induced ROS increase, necroptosis mediators and NADPH oxidase activity, thus dodging cell loss (68). Acute ethanol exposure decreases angiogenesis by reducing microvascular endothelial cell migration and tubulogenesis, as well as impairs wound healing measured by the relative epithelial gap and granulation tissue area. Interestingly, the DHA metabolite 14S, 21-diHDHA improves wound healing and increases vascularization, counteracting alcohol damage (69).

## 5. Conclusion

Alcohol elicits long-lasting body damage being one leading death cause surpassing diabetes, AIDS and tuberculosis (37). Compelling pieces of evidence have shown that n-3 intake, including maternal n-3 consumption, has beneficial effects in alcohol-elicited neurodegeneration, particularly in FASD, and alcoholic liver damage, also improving alcoholic gastric hemorrhage (67), pancreatitis (68) and angiogenesis (55) (Supplementary Table 1).

N-3, particularly EPA and DHA, and its derivatives reduce oxidative stress and inflammation, and decrease cell death in several pathological conditions (24, 33, 70, 71) through different mechanisms closely related to microglial activity. Thus, they modulate gene expression by surface and intracellular receptors, reducing pro-inflammatory cytokines and eicosanoids as well as promoting lipid mediators to resolve inflammation (72–74). Few receptors for DHA and EPA derivatives are known and most of them are highly expressed in microglial cells such as PPARs (75, 76). They also increase microglial phagocytic activity and their anti-inflammatory phenotype in various pathological conditions (77–79). The DHA rise in n-3 enriched diet promotes DHA incorporation into cell membranes. Membrane changes particularly in glial cells have consequences on pro-inflammatory receptor localization and related signaling cascades (80). Not the least, EPA lowers AA and DHA antagonizes the pro-inflammatory effects of AA products (81).

Despite the promising results described, more studies are needed in order to decipher the n-3 efficacy and dose required to alleviate the harmful effects of ethanol. Furthermore, the pathways by which n-3 PUFAs and their derivatives have their beneficial effects should be investigated in detail.

## Author contributions

PG: article idea. MS: literature search, data analysis, and original draft preparation. MS, IR-B, and PG: writing—review and editing. IR-B and PG: supervision. All authors contributed to the article and approved the submitted version.

## References

[1] OMS. *Global Status Report on Alcohol and Health 2014. Glob Status Rep Alcohol.* Geneva: WHO (2014).

[2] Thomes P Rasineni K Saraswathi V Kharbanda K Clemens D Sweeney S Natural recovery by the liver aNd other orgaNs after chroNic alcohol use. *Alcohol Res.* (2021) 41:05. 10.35946/arcr.v41.1.05 33868869 PMC8041137

[3] Clements K Smith L Reynolds J Overton P Thomas J Napper R. Early postnatal ethanol exposure: glutamatergic excitotoxic cell death during acute withdrawal. *Neurophysiology.* (2012) 44:376–86.

[4] Gursky Z Savage L Klintsova A. Executive functioning-specific behavioral impairments in a rat model of human third trimester binge drinking implicate prefrontal-thalamo-hippocampal circuitry in Fetal Alcohol Spectrum Disorders. *Behav Brain Res.* (2021) 405:113208. 10.1016/j.bbr.2021.113208 33640395 PMC8005484

[5] Broadwater M Liu W Crews F Spear L. Persistent loss of hippocampal neurogenesis and increased cell death following adolescent, but not adult, chronic ethanol exposure. *Dev Neurosci.* (2014) 36:297–305. 24993092 10.1159/000362874 PMC4125431

[6] Morris S Eaves D Smith A Nixon K. Alcohol inhibition of neurogenesis: a mechanism of hippocampal neurodegeneration in an adolescent alcohol abuse model. *Hippocampus.* (2010) 20:597–607. 10.1002/hipo.20665 19554644 PMC2861155

[7] Peñasco S Rico-Barrio I Puente N Fontaine C Ramos A Reguero L Intermittent ethanol exposure during adolescence impairs cannabinoid Type 1 receptor- dependent long-term depression and recognition memory in adult mice. *Neuropsychopharmacology.* (2020) 45:309–18. 10.1038/s41386-019-0530-5 31569197 PMC6901552

[8] Drissi I Deschamps C Fouquet G Alary R Peineau S Gosset P Memory and plasticity impairment after binge drinking in adolescent rat hippocampus: GluN2A/GluN2B NMDA receptor subunits imbalance through HDAC2. *Addict Biol.* (2019) 25:e12760. 10.1111/adb.12760 31056842

[9] Vetreno R Crews F. Binge ethanol exposure during adolescence leads to a persistent loss of neurogenesis in the dorsal and ventral hippocampus that is associated with impaired adult cognitive functioning. *Front Neurosci.* (2015) 9:35. 10.3389/fnins.2015.00035 25729346 PMC4325907

[10] Sanchez-Marin L Pavon F Decara J Suarez J Gavito A Castilla-Ortega E Effects of intermittent alcohol exposure on emotion and cognition: a potential role for the endogenous cannabinoid system and neuroinflammation. *Front Behav Neurosci.* (2017) 11:15. 10.3389/fnbeh.2017.00015 28223925 PMC5293779

[11] Rico-Barrio I Peñasco S Puente N Ramos A Fontaine C Reguero L Cognitive and neurobehavioral benefits of an enriched environment on young adult mice after chronic ethanol consumption during adolescence. *Addict Biol.* (2018) 24:969–80. 10.1111/adb.12667 30106197

[12] Rico-Barrio I Peñasco S Lekunberri L Serrano M Egaña-Huguet J Mimenza A Environmental enrichment rescues endocannabinoid-dependent synaptic plasticity lost in young adult male mice after ethanol exposure during adolescence. *Biomedicines.* (2021) 9:825. 10.3390/biomedicines9070825 34356889 PMC8301393

[13] Crews F Vetreno R Broadwater M Robinson D. Adolescent alcohol exposure persistently impact adult neurobiology and behavior. *Pharmacol Rev.* (2016) 68:1074–109. 27677720 10.1124/pr.115.012138 PMC5050442

[14] De Bellis M Clark DB Beers SR Soloff PH Boring AM Hall J Hippocampal volume in adolescent-onset alcohol use disorders.. *Am J Psychiatry.* (2000) 157:737–44. 10784466 10.1176/appi.ajp.157.5.737

[15] Sakharkar A Vetreno R Zhang H Kokare D Fulton T Pandey S A role for histone acetylation mechanisms in adolescent alcohol exposure-induced deficits in hippocampal brain-derived neurotrophic factor expression and neurogenesis markers in adulthood. *Brain Struct Funct.* (2016) 221:4691–703. 10.1007/s00429-016-1196-y 26941165 PMC5010799

[16] West R Maynard M Leasure J. Binge ethanol effects on prefrontal cortex neurons, spatial working memory and task-induced neuronal activation in male and female rats. *Physiol Behav.* (2018) 188:79–85. 10.1016/j.physbeh.2018.01.027 29407478 PMC5845786

[17] Squeglia L Jacobus J Tapert S. The influence of substance use on adolescent brain development. *Clin EEG Neurosci.* (2009) 40:31–8. 19278130 10.1177/155005940904000110 PMC2827693

[18] Montesinos J Alfonso-Loeches SG. Impact of the innate immune response in the actions of ethanol on the central nervous system. *Alcohol Clin Exp Res.* (2016) 40:2260–70. 27650785 10.1111/acer.13208

[19] Pascual M Montesinos J Guerri C. Role of the innate immune system in the neuropathological consequences induced by adolescent binge drinking. *Wiley Interdiscip Rev Membr Transp Signal.* (2017) 96:765–80. 10.1002/jnr.24203 29214654

[20] Bonilla-Del Rßo I Puente N Peñasco S Rico I Gutierrez-Rodriguez A. Adolescent ethanol intake alters cannabinoid type-1 receptor localization in astrocytes of the adult mouse hippocampus. *Addict Biol.* (2019) 24:182–92. 10.1111/adb.12585 29168269

[21] Metna-laurent M Marsicano G. Rising stars modulation of brain functions by astroglial type-1 cannabinoid receptors. *Glia.* (2015) 63:353–64. 10.1002/glia.22773 25452006

[22] Joffre C Rey C Layé S. N-3 polyunsaturated fatty acids and the resolution of neuroinflammation. *Front Pharmacol.* (2019) 10:1022. 10.3389/fphar.2019.01022 31607902 PMC6755339

[23] Calder P. Docosahexaenoic acid. *Ann Nutr Metab.* (2016) 69:8–21. 27842299 10.1159/000448262

[24] Bazan N. Neuroprotectin D1 (n.d.): a DHA-derived mediator that protects brain and retina against cell injury-induced oxidative stress. *Brain Pathol.* (2005) 15:159–66. 10.1111/j.1750-3639.2005.tb00513.x 15912889 PMC8095981

[25] Milne G Morrow J Picklo M. Elevated oxidation of docosahexaenoic acid, 22:6 (n-3), in brain regions of rats undergoing ethanol withdrawal. *Neurosci Lett.* (2006) 405:172–4. 10.1016/j.neulet.2006.06.058 16875780

[26] Strokin M Sergeeva M Reiser G. Prostaglandin synthesis in rat brain astrocytes is under the control of the n-3 docosahexaenoic acid, released by group VIB calcium-independent phospholipase A2. *J Neurochem.* (2007) 102:1771–82. 10.1111/j.1471-4159.2007.04663.x 17555549

[27] Akbar M Baick J Calderon F Wen Z Kim H. Ethanol promotes neuronal apoptosis by inhibiting phosphatidylserine accumulation. *J Neurosci Res.* (2006) 83:432–40. 10.1002/jnr.20744 16397898

[28] Tajuddin N Moon K Marshall S Nixon K Neafsey E Kim H Neuroinflammation and neurodegeneration in adult rat brain from binge ethanol exposure: abrogation by docosahexaenoic acid. *PLoS One.* (2014) 9:e101223. 10.1371/journal.pone.0101223 25029343 PMC4100731

[29] Lafourcade M Larrieu T Mato S Duffaud A Sepers M Matias I Nutritional omega-3 deficiency abolishes endocannabinoid-mediated neuronal functions. *Nat Neurosci.* (2011) 14:345–50. 10.1038/nn.2736 21278728

[30] Manduca A Bara A Larrieu T Lassalle O Joffre C Layé S Amplification of mGlu5-endocannabinoid signaling rescues behavioral and synaptic deficits in a mouse model of adolescent and adult dietary polyunsaturated fatty acid imbalance. *J Neurosci.* (2017) 37:6851–68. 10.1523/JNEUROSCI.3516-16.2017 28630250 PMC6705718

[31] Thomazeau A Bosch-Bouju C Manzoni O Layé S. Nutritional n-3 PUFA deficiency abolishes endocannabinoid gating of hippocampal long-term potentiation. *Cereb Cortex.* (2017) 27:2571–9. 10.1093/cercor/bhw052 26946127

[32] Raabe R Mathies L Davies A Bettinger J. The Omega-3 fatty acid eicosapentaenoic acid is required for normal alcohol response behaviors in *C. elegans*. *PLoS One.* (2014) 9:e105999. 10.1371/journal.pone.0105999 25162400 PMC4146551

[33] Valenzuela BR Barrera RC González-Astorga M Sanhueza CJ Valenzuela BA. Omega-3 fatty acids (EPA and DHA) and its application in diverse clinical situations. *Rev Chil Nutr.* (2011) 38:356–67.

[34] Calon F Lim G Yang F Morihara T Teter B Ubeda O Docosahexaenoic acid protects from dendritic patology in an alzheimer’s disease mouse model. *Neuron.* (2004) 43:633–45. 15339646 10.1016/j.neuron.2004.08.013 PMC2442162

[35] Mori M Delattre A Carabelli B Pudell C Bortolanza M Staziaki P Neuroprotective effect of omega-3 polyunsaturated fatty acids in the 6-OHDA model of Parkinson’s disease is mediated by a reduction of inducible nitric oxide synthase. *Nutr Neurosci.* (2018) 21:341–51. 10.1080/1028415X.2017.1290928 28221817

[36] Pérez-Matute P Pérez-Echarri N Martínez J Marti A Moreno-Aliaga M. Eicosapentaenoic acid actions on adiposity and insulin resistance in control and high-fat-fed rats: role of apoptosis, adiponectin and tumour necrosis factor-α. *Br J Nutr.* (2007) 97:389–98. 10.1017/S0007114507207627 17298710

[37] World Health Organization. *Global Status Report on Alcohol and Health 2018. Global Status Report on Alcohol and Health 2018.* Geneva: WHO (2018).

[38] Wainwright P Huang Y Mills D Ward G Ward R McCutcheon D. Interactive effects of prenatal ethanol and N-3 fatty acid supplementation on brain development in mice. *Lipids.* (1989) 24:989–97. 2615573 10.1007/BF02544067

[39] Furuya H Aikawa H Yoshida T Okazaki I. The use of docosahexaenoic acid supplementation to ameliorate the hyperactivity of rat pups induced by in utero ethanol exposure. *Environ Health Prev Med.* (2000) 5:103–10. 10.1265/ehpm.2000.103 21432193 PMC2723580

[40] Balaszczuk V Salguero J Villarreal R Scaramuzza R Mendez S Abate P. Hyperlocomotion and anxiety- like behavior induced by binge ethanol exposure in rat neonates. Possible ameliorative effects of Omega 3. *Behav Brain Res.* (2019) 372:112022. 10.1016/j.bbr.2019.112022 31181220

[41] Wellmann K George F Brnouti F Mooney S. Docosahexaenoic acid partially ameliorates deficits in social behavior and ultrasonic vocalizations caused by prenatal ethanol exposure. *Behav Brain Res.* (2015) 286:201–11. 25746516 10.1016/j.bbr.2015.02.048 PMC4392387

[42] Ward G Xing H Wainwright P. Effects of postnatal ethanol exposure on brain growth and lipid composition in n-3 fatty acid-dependent and -adequate rats. *Lipids.* (1999) 34:1777–1186. 10.1007/s11745-999-0469-2 10606040

[43] Kusat Ol K Kanbak G Oðlakcı Ilhan A Burukoglu D Yücel F. The investigation of the prenatal and postnatal alcohol exposure-induced neurodegeneration in rat brain: protection by betaine and/or omega-3. *Child’s Nerv Syst.* (2016) 32:467–74. 26732065 10.1007/s00381-015-2990-1

[44] Patten A Brocardo P Christie B. Omega-3 supplementation can restore glutathione levels and prevent oxidative damage caused by prenatal ethanol exposure. *J Nutr Biochem.* (2013) 24:760–9. 10.1016/j.jnutbio.2012.04.003 22841392

[45] Patten A Sickmann H Dyer R Innis S Christie B. Omega-3 fatty acids can reverse the long-term deficits in hippocampal synaptic plasticity caused by prenatal ethanol exposure. *Neurosci Lett.* (2013) 551:7–11. 10.1016/j.neulet.2013.05.051 23872044

[46] Rashid M Kim H. N-Docosahexaenoylethanolamine ameliorates ethanol-induced impairment of neural stem cell neurogenic differentiation. *Neuropharmacology.* (2016) 102:174–85. 10.1016/j.neuropharm.2015.11.011 26586023 PMC4698216

[47] Collins M Moon K Tajuddin N Neafsey E Kim Y. Docosahexaenoic acid (DHA) prevents binge ethanol-dependent aquaporin-4 elevations while inhibiting neurodegeneration: experiments in rat adult-age entorhino-hippocampal slice cultures. *Neurotox Res.* (2013) 23:105–10. 10.1007/s12640-012-9360-5 23184649 PMC3726012

[48] Brown IJ Achille N Neafsey E Collins M. Binge ethanol-induced neurodegeneration in rat organotypic brain slice cultures: effects of PLA2 inhibitor mepacrine and docosahexaenoic acid (DHA). *Neurochem Res.* (2009) 34:260–7. 10.1007/s11064-008-9765-y 18592376 PMC2907643

[49] Aliche-Djoudi F Podechard N Chevanne M Nourissat P Catheline D Legrand P Physical and chemical modulation of lipid rafts by a dietary n-3 polyunsaturated fatty acid increases ethanol-induced oxidative stress. *Free Radic Biol Med.* (2011) 51:2018–30. 10.1016/j.freeradbiomed.2011.08.031 21945097

[50] Aliche-Djoudi F Podechard N Collin A Chevanne M Provost E Poul M A role for lipid rafts in the protection afforded by docosahexaenoic acid against ethanol toxicity in primary rat hepatocytes. *Food Chem Toxicol.* (2013) 60:286–96. 10.1016/j.fct.2013.07.061 23907024

[51] Rolo A Teodoro J Palmeira C. Role of oxidative stress in the pathogenesis of nonalcoholic steatohepatitis. *Free Radic Biol Med.* (2012) 52:59–69. 22064361 10.1016/j.freeradbiomed.2011.10.003

[52] Feltham B Louis X Kapourchali F Eskin M Suh MDH. A supplementation during prenatal ethanol exposure alters the expression of fetal rat liver genes involved in oxidative stress regulation. *Appl Physiol Nutr Metab.* (2019) 44:744–50. 10.1139/apnm-2018-0580 30521352

[53] Wang M Zhang X Ma L Feng R Yan C Su H Omega-3 polyunsaturated fatty acids ameliorate ethanol-induced adipose hyperlipolysis: a mechanism for hepatoprotective effect against alcoholic liver disease. *Biochim Biophys Acta - Mol Basis Dis.* (2017) 1863:3190–201. 10.1016/j.bbadis.2017.08.026 28847514

[54] Huang W Wang B Li X Kang J. Endogenously elevated n-3 polyunsaturated fatty acids alleviate acute ethanol-induced liver steatosis. *BioFactors.* (2015) 41:453–62. 10.1002/biof.1246 26637972

[55] Song B Moon K Olsson N Salem N. Prevention of alcoholic fatty liver and mitochondrial dysfunction in the rat by long-chain polyunsaturated fatty acids. *J Hepatol.* (2008) 49:262–73. 10.1016/j.jhep.2008.04.023 18571270 PMC2532851

[56] Huang L Wan J Wang B He C Ma H Li T Suppression of acute ethanol-induced hepatic steatosis by docosahexaenoic acid is associated with downregulation of stearoyl-CoA desaturase 1 and inflammatory cytokines. *Prostaglandins Leukot Essent Fat Acids.* (2013) 88:347–53. 10.1016/j.plefa.2013.02.002 23474173

[57] Reyes-Gordillo K Shah R Varatharajalu R Garige M Leckey L Lakshman M. Low-ω3 fatty acid and soy protein attenuate alcohol-induced fatty liver and injury by regulating the opposing lipid oxidation and lipogenic signaling pathways. *Oxid Med Cell Longev.* (2016) 2016:1840513. 10.1155/2016/1840513 PMC5203909 28074114

[58] Wada S Yamazaki T Kawano Y Miura S Ezaki O. Fish oil fed prior to ethanol administration prevents acute ethanol-induced fatty liver in mice. *J Hepatol.* (2008) 49:441–50. 10.1016/j.jhep.2008.04.026 18620774

[59] Warner D Warner J Hardesty J Song Y Chen C Chen Z Beneficial effects of an endogenous enrichment in n3-PUFAs on Wnt signaling are associated with attenuation of alcohol- mediated liver disease in mice. *FASEB J.* (2021) 35:e21377. 10.1096/fj.202001202R 33481293 PMC8243414

[60] Hernández-Rodas M Valenzuela R Echeverría F Rincón-Cervera MÁ Espinosa A Illesca P Supplementation with docosahexaenoic acid and extra virgin olive oil prevents liver steatosis induced by a high-fat diet in mice through PPAR-α and Nrf2 upregulation with concomitant SREBP-1c and NF-kB downregulation. *Mol Nutr Food Res.* (2017) 61 1–14. 10.1002/mnfr.201700479 28940752

[61] Warner D Warner J Hardesty J Song Y King T Kang J Decreased ω-6:ω-3 PUFA ratio attenuates ethanol-induced alterations in intestinal homeostasis, microbiota, and liver injury. *J Lipid Res.* (2019) 60:2034–49. 10.1194/jlr.RA119000200 31586017 PMC6889711

[62] López-Vicario C Rius B Alcaraz-Quiles J García-Alonso V Lopategi A Titos E Pro-resolving mediators produced from EPA and DHA: overview of the pathways involved and their mechanisms in metabolic syndrome and related liver diseases. *Eur J Pharmacol.* (2016) 785:133–43. 10.1016/j.ejphar.2015.03.092 25987424

[63] Li J Deng X Bai T Wang S Jiang Q Xu K. Resolvin D1 mitigates non-alcoholic steatohepatitis by suppressing the TLR4-MyD88-mediated NF−κB and MAPK pathways and activating the Nrf2 pathway in mice. *Int Immunopharmacol.* (2020) 88:106961. 10.1016/j.intimp.2020.106961 33182038

[64] Chen X Gong X Jiang R Wang B Kuang G Li K Resolvin D1 attenuates CCl4-induced acute liver injury involving up-regulation of HO-1 in mice. *Immunopharmacol Immunotoxicol.* (2016) 38:61–7. 10.3109/08923973.2015.1115517 26630551

[65] Rius B Titos E Morán-Salvador E López-Vicario C García-Alonso V González-Périz A Resolvin D1 primes the resolution process initiated by calorie restriction in obesity-induced steatohepatitis. *FASEB J.* (2014) 28:836–48. 10.1096/fj.13-235614 24249635

[66] Kapourchali F Louis X Eskin M Suh MA. pilot study on the effect of early provision of dietary docosahexaenoic acid on testis development, functions, and sperm quality in rats exposed to prenatal ethanol. *Birth Defects Res.* (2020) 112:93–104. 10.1002/bdr2.1614 31697449

[67] Hunter B McDonald G Gibney M. The effects of acute and chronic administration of n-6 and n-3 polyunsaturated fatty acids on ethanol-induced gastric haemorrhage in rats. *Br J Nutr.* (1992) 67:501–7. 10.1079/bjn19920054 1622986

[68] Ku L Lee J Lim J Jin L Seo J Kim H. Docosahexaenoic acid inhibits ethanol/palmitoleic acid-induced necroptosis in AR42J cells. *J Physiol Pharmacol.* (2020) 71:437–50. 10.26402/jpp.2020.3.15 33077696

[69] Tian H Lu Y Shah S Hong S. Novel 14S,21-dihydroxy-docosahexaenoic acid rescues wound healing and associated angiogenesis impaired by acute ethanol intoxication/exposure. *J Cell Biochem.* (2010) 111:266–73. 10.1002/jcb.22709 20506249 PMC3308707

[70] Gómez-Soler M Cordobilla B Morató X Fernández-Dueñas V Domingo J Ciruela F. Triglyceride form of docosahexaenoic acid mediates neuroprotection in experimental parkinsonism. *Front Neurosci.* (2018) 12:604. 10.3389/fnins.2018.00604 30233293 PMC6127646

[71] Bousquet M Saint-Pierre M Julien C Salem N Cicchetti F Calon F. Beneficial effects of dietary omega-3 polyunsaturated fatty acid on toxin-induced neuronal degeneration in an animal model of Parkinson’s disease. *FASEB J.* (2008) 22:1213–25. 10.1096/fj.07-9677com 18032633

[72] Serhan C. Pro-resolving lipid mediators are leads for resolution physiology. *Nature.* (2014) 510:92–101. 24899309 10.1038/nature13479 PMC4263681

[73] Calder P. n-3 Polyunsaturated fatty acids, inflammation, and inflammatory diseases. *Am J Clin Nutr.* (2006) 83(Suppl. 6):1505S–19S. 16841861 10.1093/ajcn/83.6.1505S

[74] Calder P. Fatty acids and inflammation: the cutting edge between food and pharma. *Eur J Pharmacol.* (2011) 668(Suppl. 1):S50–8. 10.1016/j.ejphar.2011.05.085 21816146

[75] Serhan CN Krishnamoorthy S Recchiuti A Chiang N. Novel anti-inflammatory-pro-resolving mediators and their receptors. *Curr Top Med Chem.* (2011) 11:629–47. 21261595 10.2174/1568026611109060629 PMC3094721

[76] Zhang Y Chen K Sloan S Bennett M Scholze A O’Keeffe S An RNA-sequencing transcriptome and splicing database of glia, neurons, and vascular cells of the cerebral cortex. *J Neurosci.* (2014) 34:11929–47. 10.1523/JNEUROSCI.1860-14.2014 25186741 PMC4152602

[77] Ebert S Weigelt K Walczak Y Drobnik W Mauerer R Hume D Docosahexaenoic acid attenuates microglial activation and delays early retinal degeneration. *J Neurochem.* (2009) 110:1863–75. 10.1111/j.1471-4159.2009.06286.x 19627444

[78] Hjorth E Zhu M Toro V Vedin I Palmblad J Cederholm T Omega-3 fatty acids enhance phagocytosis of alzheimer’s disease-related amyloid-β42 by human microglia and decrease inflammatory markers. *J Alzheimer’s Dis.* (2013) 35:697–713. 23481688 10.3233/JAD-130131

[79] Chen S Zhang H Pu H Wang G Li W Leak R N-3 PUFA supplementation benefits microglial responses to myelin pathology. *Sci Rep.* (2014) 4:7458. 10.1038/srep07458 25500548 PMC4264015

[80] Layé S Nadjar A Joffre C Bazinet R. Anti-inflammatory effects of omega-3 fatty acids in the brain: physiological mechanisms and relevance to pharmacology. *Pharmacol Rev.* (2018) 70:12–38. 10.1124/pr.117.014092 29217656

[81] Farooqui A. *Lipid Mediators and their Metabolism in the Brain.* New York, NY: Springer (2011).

