# Supplementary material for: The effect of omega-3 fatty acids on alcohol-induced damage
Source: Front Nutr. 2023 Apr 5;10:1068343. doi: 10.3389/fnut.2023.1068343 (PMC10113533; doi:10.3389/fnut.2023.1068343)
Supplement: Supplementary file 1 [file Table_1.DOCX]

Supplementary Material

Supplementary Table 1. N-3 effects on alcohol-induced damage.

| STUDY | ANIMAL/CELL MODEL | ETHANOL | N-3 | N-3 MAIN FINDINGS |
| --- | --- | --- | --- | --- |
| Tajuddin et al. [28] | Adult SD rat and 60±3 days HEC | Intragastric; initial dose of 5g/kg and then adjusted, every 8h, 4 days  HEC: 100mM, 16h exposed, 4 days | HEC: 25-50µM DHA, 4h before EtOH and during 4 days | Inhibition of neurodegeneration and oxidative stress |
| Wainwright PE et al. [38] | Pregnant B6D2F1 mice | Liquid diet: 5 % EDC on GD5, 10 % EDC on GD6 and GD17, 20 % on GD7-16 | 10 % SF +10 % polepa (15.24 % n-3 of FA) vs. control 20% SF on GD5-17 | Recovery of decreased brain and body weight and increased n-6/n-3 |
| Furuya H et al. [39] | Pregnant Wistar rats | 10 % EtOH on GD7-PW4 | 3 % SF and 2 % DHA mixture vs. control 5% SF on GD7-PW10 | Improvement of increased n-6/n-3, locomotion and anxiety |
| Balaszczuk V et al. [40] | Wistar rats | Subcutaneous; 2 dosage of 2.5g/kg, 2h apart on PD7 | Intragastric; 720mg/kg n-3 15min after EtOH | Improvement of increased locomotion and anxiety |
| Wellmann KA et al. [41] | Pregnant Long-Evans rats | Liquid diet; 11.5% EDC on GD6-7, 23.5% EDC on GD8-10, 35% EDC on GD11-21 | Intragastric; 10g/kg DHA on PD11-20 | Improvement of social behavior and ultrasonic vocalizations |
| Ward GR et al. [42] | Pregnant SD rats | Gastrostomy; 7 % (v/v) 4 consecutive feedinds/day on PD6-9 | Gastrostomy; LA and LNA (chow 5.08 %; milk 3.99 %) diet vs. LA diet on dams PD5-pups PD9 | Improvement of increased n-6/n-3 |
| Kusat K et al. [43] | Pregnant SD rats | Liquid diet; 2.4% EtOH on GD6-8, 4.8% EtOH on GD9-11, 7.2% EtOH on GD11-PD21 | 30mg n-3/100g/day on GD3-PD21 | Decrease neurodegeneration and oxidative stress |
| Patten AR et al. [44] and [45] | Pregnant SD rats | Liquid diet; 12 % EDC on GD1, 24 % EDC on GD2, 35.5 % EDC on GD3-GD21 | 34.2 % n-3 (24.6 % DHA) vs. 0.29 % n-3 control on PD0-21 via dams milk and PD22-70 chow diet | *[25]*Decrease brain oxidative stress. *[26]*Recovery of male hippocampal LTP reduction |
| Rashid MA et al. [46] | Fetal Wistar rat NSC | 25-50mM, 15min, 1h, 6h and 4 days | Synaptamide bound to 0.05 % (wt/vol) BSA, 3-4 days | Improvement of neurogenic differentiation |
| Collins MA et al. [47] | 62±3 days HEC | 100mM, 16h exposed, 4 days | 25-50µM DHA, 4h before EtOH and during 4 days | Inhibition of neurodegeneration and oxidative stress |
| Brown III J et al. [48] | 24±1 days HEC | 100mM EtOH, 24h for 3 days, 12-14h daily next 2 days and 24h next day | 25µM DHA, 3 hours before EtOH and during 6 days | Inhibition of neurodegeneration and oxidative stress |
| Aliche-Djoudi F et al. [49] | Adult SD rat hepatocytes | 50mM EtOH for 1,1.5 or 5h, after EPA | 200µM EPA for 18h | Promotion of oxidation and cell death |
| Aliche-Djoudi F et al. [50] | Adult SD rats hepatocytes | 50mM EtOH for 1, 1.5 or 5h, after DHA | 200µM DHA for 18h | Prevention of oxidation and cell death |
| Felthman BA et al. [52] | Pregnant SD rats | Intragastric; twice/day, 1g/kg 3 days prior to mating, 2g/kg 2 days prior to mating and 3g/kg since then until GD10 | 1.4 % (w/w) DHA of FA on GD1-20 | Reduction of liver enlargement and oxidative stress |
| Wang M et al. [53] | *Fat-1* and C57BL/6 mice | Liquid diet; 28 % EDC for 10 days and intragastric; last dose of 5g/kg BW | 19.8g/L EPA+DHA for WT mice | Reduction of liver n-6/n-3 and inflammation |
| Huang W et al. [54] | *Fat-1* and C57BL/6J mice | Intragastric; 4.7g/kg 3 times, 12h apart on PW9 | AIN-76A with 10 % corn oil (n-6 rich) | Reduction of liver n-6/n-3, oxidative stress and inflammation |
| Song BJ et al. [55] | Long-Evan rats | Liquid diet; 36 % EDC | 0.5 % DHA derived calories | Reduction of liver steatosis and oxidative stress |
| Huang LL et al. [56] | C57BL/6J mice | Intragastric; 4.7g/kg 3 times, 12h apart on PW9 | Intragastric, 250mg/kg BW DHA with alcohol | Reduction of liver n-6/n-3, oxidative stress and inflammation |
| Reyes-Gordillo K et al. [57] | Wistar rats | Liquid diet; 36 % EDC for 4 weeks | 14.1 % vs. 2.7 % n-3 (fish oil) derived calories for 4 weeks | Reduction of liver steatosis |
| Wada S et al. [58] | C57BL/6J mice | Intragastric; 3g/kg one dose at PW8 | 13g/100g fish oil (7 % EPA, 24 % DHA) vs. SF day prior to EtOH | Reduction of liver TG accumulation |
| Warner DR et al. [59] and [61] | *Fat-1* and C57BL/6J mice | Liquid diet; EtOH (v/v) 2 days each of 1 %, and 2 %, 1 week each for 4 % and 5 %, and finally, 6 % for 3 weeks. | F1259SP diet | *[46]*Reduction of liver steatosis, n-6/n-3 and inflammation *[47]*Reduction of liver injury |
| Kapourchali FR et al. [66] | Pregnant SD rats | Intragastric; twice/day, 1g/kg 3 days prior to mating, 2g/kg 2 days prior to mating and 3g/kg since then until pup birth | 1.4 % (w/w) DHA of FA on GD0-PD90 | Improvement of reduced testosterone production and level |
| Hunter B et al. [67] | Wistar rats | Oral; 2 ml absolute EtOH | 100 g/kg of fish oil for 5 weeks or 1h before EtOH 1 ml of a solution with 120 mM-EPA | Reduction of the stomach lesion by fish oil diet but not by acute EPA administration |
| Ku L et al. [68] | Rat pancreatic acinar AR42J cells | 150 mM EtOH and 50 μM palmitoleic acid for 10min, 15min or 6h | 1 or 2 μM DHA for 1 h prior to EtOH | Reduction of ROS and cell death |
| Tian H et al. [69] | 8-10 weeks old C57BL/6J mice  mMVEC | Injection; 150 μl 20 % EtOH | Intradermally injected; 50ng 14S,21-diHDHA | Restored wound healing |

^BSA: bovine serum albumin; EDC: ethanol derived calories; EtOH: ethanol; GD: gestational day; HEC: hippocampal-entorhinal cortical slice cultures; mMVEC: murine microvascular endothelial cells; LA: linoleic acid; LNA: linolenic acid; PD: postnatal day; PW: postnatal week; SD: Sprague-Dawley; SF: safflower; TG: triglycerides^
